# Impact of the COVID-19 pandemic on the mental health of health care workers in CYPRUS

**DOI:** 10.1192/j.eurpsy.2021.742

**Published:** 2021-08-13

**Authors:** A. Chatzittofis, M. Karanikola, K. Michailidou, A. Constantinidou

**Affiliations:** 1 Medical School, University of Cyprus, Nicosia, Cyprus; 2 Department Of Nursing, Cyprus University of Technology, Limassol, Cyprus; 3 Biostatistics Unit, The Cyprus Institute of Neurology & Genetics, Nicosia, Cyprus

**Keywords:** ptsd, health care workers, covid 19, Depression

## Abstract

**Introduction:**

The coronavirus disease 2019 (COVID-19) has a great impact on health care workers (HCWs) who are exposed to high levels of stress and trauma leading to negative mental health outcomes, including stress-related symptoms and depressive symptoms.

**Objectives:**

The aim of this study was to investigate the prevalence of depressive symptoms, anxiety and post traumatic stress symptoms related to to the COVID 19 pandemic in Cyprus.

**Methods:**

In this cross-sectional study, we report on mental health outcomes among HCWs in Cyprus. Data were collected between May 3rd and May 27th, 2020, using an online questionnaire that included demographics, the 9-item Patient Health Questionnaire (PHQ-9), assessing depressive symptoms, the Impact of Events Scale Revised (IES-R) measuring PTSD symptoms and the 10 item Perceived Stress Scale (PSS) measuring stress.

**Results:**

424 Health Care Workers (HCWs) participated in the study. 79 HCWs (18,6%) scored in PHQ-9 above the cut-off for depression while 62 HCWs (14,6%) scored high enough in IES-R indicating a diagnosis of post-traumatic stress disorder. The prevalence of depression and PTSD symptoms were significantly higher among nurses compared to doctors and other HCWs. (20.7% versus 10.8%; adjusted prevalence ratio, 1.70 [95% CI, 1.06 to 2.73]), after adjustment for age and sex.

**Conclusions:**

Even in countries like Cyprus with minimum impact of the COVID-19 pandemic, the impact on the mental health of HCWs is substantial with nurses being more vulnerable.

